# Anesthetic Considerations in Geriatric Patients Undergoing Cataract Surgery: A Review of Perioperative Management and Outcomes

**DOI:** 10.7759/cureus.105829

**Published:** 2026-03-25

**Authors:** Saul J Prado Fonseca, Fabiana M Araya Padilla, Nicole Rodríguez, Gabriel Anchía Jiménez, Jose Ignacio Chacon Soto, Natalia Ruiz Rojas

**Affiliations:** 1 Emergency Medicine, Hospital Monseñor Sanabria Martínez, Puntarenas, CRI; 2 General Medicine, Caja Costarricense de Seguro Social (CCSS), Alajuela, CRI; 3 General Medicine, Universidad de Costa Rica (UCR) Facultad de Medicina, San Pedro, CRI

**Keywords:** anesthetic techniques, cataract surgery, geriatric anesthesia, perioperative management, physiological aging, sedation safety

## Abstract

Cataract surgery is one of the most frequently performed procedures worldwide and is particularly common in elderly patients due to the increased prevalence of age-related visual impairment. However, aging is associated with physiological, pharmacological, and clinical changes that significantly influence anesthetic management and perioperative outcomes. Age-related alterations in cardiovascular function, including reduced cardiac reserve, increased arterial stiffness, and impaired autonomic regulation, increase susceptibility to intraoperative hypotension and hemodynamic instability. Respiratory changes such as decreased lung elasticity, reduced vital capacity, and impaired gas exchange further contribute to the risk of hypoventilation and hypoxemia, particularly during sedation. In addition, neurological changes and increased sensitivity to sedatives and opioids elevate the risk of postoperative cognitive dysfunction and delirium. Comprehensive preoperative evaluation is essential for minimizing perioperative risk in elderly patients. This assessment should include a detailed medical history, identification of comorbidities such as hypertension, diabetes, and renal impairment, cardiovascular and respiratory evaluation, and careful review of the patient’s medication regimen. Functional and cognitive assessments also play an important role in predicting recovery and guiding perioperative planning. In most cases, cataract surgery is performed under topical or regional anesthesia, which offers advantages such as rapid recovery and fewer systemic complications compared with general anesthesia. Intravenous sedation may be used selectively to improve patient comfort, although careful dosing is required due to increased pharmacodynamic sensitivity in elderly individuals. Perioperative complications in this population include cardiovascular instability, respiratory depression, prolonged sedation, and postoperative delirium. Strategies to optimize outcomes include careful preoperative optimization, individualized anesthetic selection, continuous intraoperative monitoring, and a multidisciplinary perioperative approach.

## Introduction and background

Preoperative assessment and optimization play a fundamental role in reducing perioperative risk in elderly patients undergoing cataract surgery. Older adults frequently present with multiple chronic conditions, particularly cardiovascular disease, which can significantly increase surgical risk and influence perioperative management. In this context, frailty has emerged as an important predictor of adverse outcomes. Therefore, the preoperative evaluation should extend beyond identification of comorbidities and include a comprehensive assessment of the patient’s overall health status and degree of frailty, allowing clinicians to better anticipate potential complications and tailor perioperative strategies accordingly. In addition to comorbidities and frailty, polypharmacy represents another critical consideration in the geriatric population. The high prevalence of multiple concurrent medications increases the likelihood of drug interactions and adverse pharmacological effects. Consequently, a thorough review of the patient’s medication regimen is essential during the preoperative assessment to identify drugs that may interact with anesthetic agents or influence perioperative stability [1].

In terms of anesthetic management, cataract surgery is most often performed using local or topical anesthesia, approaches that are generally well tolerated by older adults and are associated with fewer systemic complications compared with general anesthesia. The selection of the anesthetic technique should be individualized, considering the patient’s underlying medical conditions as well as the preferences and experience of the operating surgeon. This individualized approach allows the anesthetic plan to be adapted to the patient’s clinical profile while maintaining procedural safety and efficiency. Alongside the choice of anesthesia, sedation practices during cataract surgery can vary considerably among practitioners. Some ophthalmologists prefer minimal sedation or even the absence of sedation to reduce the risk of respiratory or cardiovascular complications. Accordingly, decisions regarding sedation should be carefully individualized, keeping both the patient’s overall health status and the anticipated complexity of the surgical procedure in mind [2].

Effective intraoperative and postoperative management are also essential components of safe surgical care in elderly patients. Continuous monitoring of vital signs during the procedure is crucial for the early detection and prompt management of potential hemodynamic instability. The implementation of minimally invasive monitoring strategies may further contribute to enhancing patient safety while maintaining comfort during the procedure [3]. Following surgery, elderly patients may experience slower recovery and an increased risk of postoperative complications compared with younger individuals. For this reason, careful postoperative monitoring and appropriate management of pain and other postoperative symptoms are necessary to facilitate a stable recovery and minimize the likelihood of adverse events [1].

Additional considerations arise when cataract surgery is performed in very old patients, particularly those over 90 years of age. In this population, surgical procedures may present greater technical challenges, including the need for higher phacoemulsification energy and an increased incidence of intraoperative complications such as floppy iris syndrome. As a result, these patients may benefit from procedures performed by experienced surgeons and from the use of tailored surgical techniques designed to address the specific anatomical and physiological characteristics associated with advanced age. Age itself has been identified as an independent factor influencing postoperative visual outcomes. Consequently, expectations regarding improvements in visual acuity should be carefully discussed with patients and aligned with their age, baseline ocular condition, and overall health status to ensure realistic postoperative goals [4]. 

The objective of this article is to review the current evidence on anesthetic considerations in geriatric patients undergoing cataract surgery, focusing on age-related physiological changes, preoperative assessment and optimization, anesthetic techniques, pharmacological considerations, perioperative management, and factors influencing surgical safety and postoperative outcomes in elderly patients.

## Review

Methodology

This was developed as a structured narrative review aimed at providing an updated and clinically integrated analysis of anesthetic considerations in geriatric patients undergoing cataract surgery, with particular emphasis on preoperative assessment, age-related physiological changes, anesthetic techniques, perioperative safety, and postoperative outcomes. The review was conducted in accordance with the Scale for the Assessment of Narrative Review Articles (SANRA) framework and followed a predefined methodological approach established prior to literature screening. Given the clinical heterogeneity of elderly patients, including variability in comorbidity burden, frailty, functional status, and anesthetic practices across institutions, a narrative interpretative synthesis was selected over quantitative pooling to integrate physiological, pharmacological, surgical, and perioperative considerations into a clinically applicable framework [5].

To improve transparency, the methodology was structured to clearly describe the processes of literature identification, selection, and synthesis, while acknowledging that, as a narrative review, it does not aim for full reproducibility comparable to systematic reviews. The objective was to provide a structured synthesis capable of supporting individualized perioperative decision-making in geriatric ophthalmic surgery.

A comprehensive literature search was conducted in PubMed, Scopus, and Web of Science, including peer-reviewed articles published in English or Spanish between January 2020 and December 2026. The final search was conducted in December 2026. This timeframe was selected to capture contemporary advances in geriatric perioperative assessment, evolving anesthetic strategies in ambulatory ophthalmic surgery, updated evidence on sedation and monitoring practices, and recent data regarding outcomes in older and very old patients undergoing cataract extraction. Foundational studies were incorporated when necessary to contextualize physiological changes associated with aging, traditional anesthetic approaches, or the historical evolution of perioperative care in cataract surgery.

The search strategy combined Medical Subject Headings (MeSH) terms and free-text keywords using Boolean operators related to cataract surgery, geriatric patients, elderly, aging, frailty, preoperative assessment, polypharmacy, topical anesthesia, local anesthesia, regional anesthesia, sedation, general anesthesia, perioperative complications, postoperative recovery, and visual outcomes. Searches were conducted in titles, abstracts, and indexed subject headings to maximize sensitivity.

The initial search yielded 118 records. After removal of duplicates, 79 articles remained for title and abstract screening. Of these, 58 underwent full-text evaluation, and 28 studies were included in the final synthesis. Study selection was performed independently by two authors, with disagreements resolved through discussion and consensus. Tge flowchart for the study process is shown in Fugure 1.

**Figure 1:**
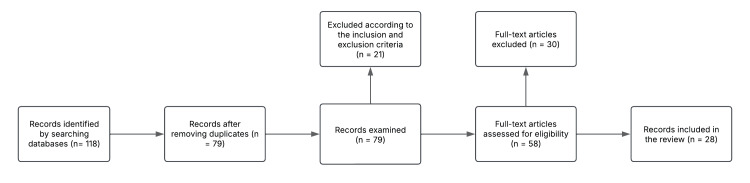
Flowchart of the process of identifying, selecting and eligibility of studies

Eligible studies included randomized controlled trials, observational cohort studies, systematic reviews, meta-analyses, expert consensus statements, and contemporary international guidelines from anesthesiology, ophthalmology, geriatric medicine, and perioperative care societies. Priority was assigned to multicenter investigations, studies with clearly defined geriatric populations, and research evaluating anesthetic safety, hemodynamic stability, sedation-related events, postoperative recovery, and visual or functional outcomes. Extracted variables included study design, patient age, comorbidity profile, frailty status, anesthetic technique, use of sedation, intraoperative monitoring practices, perioperative complications, recovery characteristics, and reported postoperative outcomes.

Data were synthesized qualitatively using an interpretative narrative approach. No formal quantitative synthesis or meta-analysis was performed because of heterogeneity in study designs, patient populations, anesthetic techniques, and reported outcomes. Methodological quality and internal validity were assessed narratively by considering risk of bias, sample size, follow-up duration, consistency of perioperative outcome definitions, and reproducibility of reported findings. In cases of conflicting evidence, greater interpretative weight was assigned to higher-level evidence and guideline-supported recommendations.

Reference lists of included studies were manually screened to identify additional relevant publications. Exclusion criteria comprised non-peer-reviewed publications, isolated case reports, editorials without outcome data, studies focused exclusively on surgical ophthalmologic technique without anesthetic relevance, redundant datasets, and studies not directly addressing perioperative assessment, anesthetic management, complications, or outcomes in geriatric patients undergoing cataract surgery. Given its narrative design, this review is subject to potential selection bias and does not aim to achieve the full reproducibility expected of systematic reviews. Artificial intelligence-based tools were used exclusively to assist in literature organization and structural coherence, whereas critical appraisal, synthesis, and final interpretation were conducted independently by the authors to preserve methodological rigor.

Age-related physiological changes and their impact on anesthesia

Aging is associated with multiple physiological changes that significantly influence anesthetic management in elderly patients. Among the most relevant alterations are those affecting the cardiovascular system. With advancing age, cardiac reserve progressively declines, leading to reductions in cardiac output and a diminished capacity to respond to physiological stress. This reduction in compensatory capacity increases the susceptibility of older adults to perioperative cardiovascular complications [1,6]. In addition to reduced cardiac reserve, aging is accompanied by increased arterial stiffness, a process that contributes to higher systolic blood pressure and increased afterload. These hemodynamic alterations may complicate intraoperative cardiovascular management during anesthesia [1]. Furthermore, autonomic regulation becomes less efficient with age, resulting in impaired autonomic responses that predispose elderly patients to hypotension and hemodynamic instability during surgical procedures. This vulnerability may be further exacerbated by the administration of certain anesthetic agents. For example, the use of propofol has been associated with increased susceptibility to hypotension in older patients, whereas alternative agents such as remimazolam may provide a more stable hemodynamic profile during anesthesia [7,8].

In parallel with cardiovascular changes, aging also produces significant alterations in respiratory physiology that can influence perioperative risk. One of the primary changes is the reduction in lung elasticity, which leads to decreased pulmonary compliance and may impair effective ventilation during anesthesia. This loss of elasticity increases the risk of complications such as atelectasis. In addition, older adults experience a reduction in vital capacity, which limits respiratory reserve and makes them more vulnerable to hypoventilation, particularly when sedative medications are administered. Age-related changes in alveolar structure and function also contribute to alterations in gas exchange, potentially resulting in impaired oxygenation and an increased risk of hypoxemia during surgical procedures. The administration of sedatives and opioids may further depress respiratory function, which underscores the need for careful monitoring and appropriate dose adjustments in this patient population [6].

Neurological changes associated with aging represent another important consideration in anesthetic management. Cognitive decline is relatively common among older adults and may influence a patient’s ability to fully understand and consent to medical procedures. Moreover, these changes are associated with an increased risk of postoperative cognitive dysfunction [1,5]. Elderly patients also demonstrate increased sensitivity to sedatives and opioid medications, often requiring lower doses of anesthetic agents to achieve the desired clinical effect. Adjusting drug doses accordingly may help reduce the likelihood of adverse effects associated with excessive sedation [9,10]. In addition, older adults have a higher risk of developing postoperative delirium, a complication that may significantly affect recovery and overall outcomes. Strategies aimed at minimizing this risk include avoiding unnecessary polypharmacy and ensuring adequate postoperative pain control [1,6].

In addition to physiological and neurological changes, aging is associated with important pharmacological alterations that influence the response to anesthetic agents. Age-related modifications in pharmacokinetics and pharmacodynamics can affect drug metabolism, distribution, and elimination, leading to greater variability in drug response among elderly patients [6,10]. As a result, anesthetic drugs may produce stronger or more prolonged effects than expected. This variability requires careful titration of anesthetic agents and individualized dosing strategies to avoid excessive sedation and prolonged recovery times [11].

Preoperative evaluation and risk stratification

A comprehensive preoperative evaluation is essential for optimizing perioperative safety in elderly patients undergoing cataract surgery. The process begins with a detailed general clinical evaluation, in which obtaining a complete medical history is fundamental. A thorough review of the patient’s medical background allows clinicians to identify previous medical conditions, prior surgical interventions, and any history of adverse reactions to anesthesia. This information is critical for anticipating potential complications and guiding the development of appropriate perioperative management strategies [12]. In addition to reviewing past medical events, it is also important to identify relevant comorbidities, as elderly patients frequently present with chronic conditions that may influence both surgical outcomes and anesthetic management. Among the most common comorbidities observed in this population are diabetes mellitus, hypertension, and renal impairment, all of which require careful consideration during perioperative planning [1].

Cardiovascular assessment represents another key component of the preoperative evaluation. Proper control of hypertension is particularly important, as poorly managed blood pressure can increase the risk of intraoperative and postoperative cardiovascular events. Similarly, the presence of coronary artery disease or heart failure requires careful evaluation and optimization prior to surgery to minimize the risk of perioperative cardiac complications. In addition, the identification and appropriate management of cardiac arrhythmias before surgery are essential steps that can help reduce the likelihood of adverse cardiac events during the procedure [1,12].

Respiratory assessment is also necessary in elderly patients, particularly because age-related changes and chronic respiratory conditions may complicate anesthetic management. Chronic obstructive pulmonary disease is one of the most common respiratory disorders in older adults and can significantly affect both intraoperative ventilation and postoperative recovery. For this reason, preoperative optimization of respiratory function is recommended in patients with this condition [1]. Evaluating potential ventilatory limitations allows clinicians to anticipate respiratory challenges and plan appropriate anesthetic and postoperative care strategies [12].

A detailed review of the patient’s medication regimen is another essential element of preoperative assessment in the geriatric population. Particular attention should be given to anticoagulants and antiplatelet agents, as these medications require careful perioperative management to balance the risks of bleeding and thromboembolic events [13]. Antihypertensive medications may also require adjustment to help maintain hemodynamic stability during surgery. In patients with diabetes, appropriate management of hypoglycemic agents is necessary to prevent perioperative hyperglycemia or hypoglycemia. Additionally, sedatives and anxiolytics should be carefully reviewed because their use may contribute to excessive sedation and respiratory depression during the perioperative period [1,12].

Beyond medical conditions and medications, evaluating the patient’s functional and cognitive status is particularly important in elderly individuals. Assessing the level of functional independence provides valuable information regarding the patient’s baseline condition and can help predict postoperative recovery and the level of care that may be required after surgery. Similarly, identifying cognitive impairment is essential for both anesthetic planning and postoperative management, as cognitive deficits may increase the risk of complications such as postoperative delirium [14]. Anxiety or an inability to cooperate during the procedure should also be recognized during the preoperative evaluation, as addressing these factors may improve patient cooperation and contribute to better surgical outcomes [1].

Risk stratification tools play an important role in guiding perioperative decision-making in elderly patients. The American Society of Anesthesiologists (ASA) physical status classification remains one of the most widely used systems for identifying high-risk patients and tailoring perioperative care accordingly [13]. In addition, other assessment tools, such as the PIRATE (Pre-Interventional Risk Assessment in The Elderly) score, may assist clinicians in stratifying perioperative risk and supporting clinical decision-making in older adults undergoing cataract surgery [15].

Anesthetic techniques in cataract surgery

Topical anesthesia represents one of the most commonly used anesthetic approaches in cataract surgery. This technique involves the application of local anesthetic agents directly to the ocular surface, allowing adequate analgesia during the procedure while avoiding invasive injections. One of the principal advantages of topical anesthesia is its association with rapid postoperative recovery and minimal systemic effects, characteristics that make it particularly suitable for outpatient surgical settings [16,17]. Despite these benefits, topical anesthesia has certain limitations, particularly in elderly patients who may have cognitive impairment or difficulty maintaining adequate cooperation during surgery. Because the technique relies heavily on patient cooperation to ensure immobility and surgical precision, its use may be challenging in individuals with significant cognitive dysfunction [18].

In addition to topical anesthesia, regional anesthesia remains an important option in cataract surgery. Techniques such as retrobulbar and peribulbar blocks involve the injection of anesthetic agents either near the optic nerve or within the tissues surrounding the eye. These approaches provide both analgesia and ocular akinesia, which can facilitate surgical conditions by reducing eye movement during the procedure. However, regional anesthetic techniques have been associated with higher complication rates compared with topical anesthesia, particularly in patients receiving anticoagulant therapy, where the risk of hemorrhagic complications may be increased. As an alternative to these sharp-needle techniques, the sub-Tenon block has been increasingly utilized. This method involves the injection of anesthetic into the sub-Tenon space and has been associated with a lower risk of complications, making it a safer option in certain clinical scenarios [16,17].

Intravenous sedation may also be used as an adjunct to local or regional anesthesia to enhance patient comfort during cataract surgery. The primary objective of sedation is to reduce anxiety and improve patient tolerance of the procedure. Commonly used agents include benzodiazepines and short-acting hypnotic medications such as propofol. In particular, the administration of low-dose propofol has been shown to reduce intraoperative pain and anxiety while improving patient satisfaction, without producing significant systemic complications when carefully titrated [19].

Although rarely required, general anesthesia may be necessary in selected cases. This approach is typically reserved for patients who are unable to cooperate with the procedure due to severe cognitive impairment, neurological disorders, or extreme anxiety that cannot be adequately managed with local anesthesia and sedation [18]. Nevertheless, the use of general anesthesia in elderly patients must be approached with caution because it is associated with higher perioperative risks compared with less invasive anesthetic techniques [7].

When comparing anesthetic approaches, both topical and regional techniques present specific advantages and limitations. Although topical anesthesia offers a less invasive method with rapid recovery, it has been associated with a higher risk of certain complications, including posterior capsule rupture and endophthalmitis, when compared with regional anesthetic techniques [17]. Consequently, the selection of the most appropriate anesthetic method should be individualized based on patient-specific factors. In geriatric patients, comorbidities often play a central role in this decision-making process. For example, patients with significant cardiovascular disease may benefit from regional anesthesia to minimize systemic effects and maintain greater hemodynamic stability during the procedure [6].

Pharmacological considerations in elderly patients

Pharmacological considerations play a critical role in the anesthetic management of elderly patients, as aging is associated with significant alterations in both pharmacokinetics and pharmacodynamics. These changes influence how drugs are distributed, metabolized, and eliminated, and they may also modify the physiological response to anesthetic agents. One of the most relevant pharmacokinetic changes in older adults involves alterations in drug distribution. In elderly patients, an increased percentage of body fat combined with decreased lean body mass results in a larger volume of distribution for lipid-soluble drugs. Consequently, the elimination half-life of these medications may be prolonged, which can lead to longer drug effects and requires careful monitoring and appropriate dose adjustments [20].

In addition to altered drug distribution, hepatic metabolism is also affected by aging. Older adults commonly experience reductions in hepatic blood flow and liver mass, factors that contribute to decreased metabolic capacity for drugs processed through hepatic pathways. This reduction in metabolic activity may prolong the pharmacological effects of certain medications and increase the risk of drug accumulation and toxicity. Renal clearance is similarly affected by age-related physiological decline. Progressive reductions in renal function can impair the elimination of drugs that are primarily excreted through the kidneys. Consequently, dose adjustments are often required in order to prevent drug accumulation and toxicity, particularly when medications with narrow therapeutic windows are used [20].

Alongside these pharmacokinetic changes, aging also produces important pharmacodynamic alterations. Elderly patients frequently exhibit increased sensitivity to sedative and analgesic medications, which can heighten the risk of oversedation and respiratory depression if standard adult doses are administered. For this reason, lower initial doses and careful titration of anesthetic agents are generally recommended in this population [20]. Moreover, the central nervous system of older adults tends to be more susceptible to the effects of anesthetic medications. This increased vulnerability may contribute to complications such as postoperative cognitive dysfunction and delirium. In response to this concern, certain anesthetic agents, including ketamine and dexmedetomidine, have been investigated for their potential neuroprotective effects in elderly patients [21,22].

Several medications are commonly used during cataract surgery, and their use requires consideration in the geriatric population. Midazolam, a benzodiazepine frequently used for sedation, has been widely utilized in ophthalmic procedures; however, its administration in older adults remains controversial because increased sensitivity to the drug may elevate the risk of cognitive impairment and prolonged sedation [23]. Propofol is another commonly used anesthetic agent, particularly for induction and sedation, but elderly patients often require dose reductions due to increased sensitivity and decreased clearance of the drug [9]. Opioid analgesics such as fentanyl are also used in certain cases to provide analgesia, although careful dosing is essential to avoid respiratory depression, which may occur more readily in older individuals [20]. Dexmedetomidine has gained attention as an alternative sedative agent because it provides both sedative and analgesic effects while producing minimal respiratory depression, making it a potentially favorable option for elderly patients undergoing cataract surgery [21,22].

Given the pharmacokinetic and pharmacodynamic changes associated with aging, dose adjustments are a central component of safe anesthetic management in older adults. Lower initial doses of anesthetic agents are generally recommended because elderly patients demonstrate increased sensitivity and altered drug metabolism [10]. In addition to starting with reduced doses, careful titration and continuous monitoring are essential to prevent oversedation and related complications, including respiratory depression and cognitive dysfunction [20].

Perioperative complications and risk factors

Cardiovascular complications represent an important concern in elderly patients undergoing surgical procedures, particularly because aging is associated with reduced physiological reserve and a higher prevalence of cardiovascular disease. One of the most frequently encountered complications in this population is intraoperative hypotension. Older patients are especially vulnerable to decreases in blood pressure during surgery, and such episodes may compromise organ perfusion and increase the risk of perioperative complications. In this context, continuous infusion of norepinephrine has been shown to effectively prevent hypotension in older patients undergoing surgery under spinal anesthesia with propofol sedation. This strategy has also been associated with a reduced requirement for intravenous fluid administration and increased urine output, suggesting improved hemodynamic stability during the procedure [8].

In addition to hypotension, cardiac arrhythmias constitute another relevant cardiovascular complication in geriatric patients. The risk of arrhythmias is particularly elevated in individuals with pre-existing cardiac conditions, such as atrial fibrillation. Several risk assessment tools have been developed to estimate the likelihood of major adverse cardiac events in surgical patients. Among these, the Revised Cardiac Risk Index and the Geriatric Sensitive Cardiac Risk Index have been used to evaluate perioperative cardiovascular risk, although their predictive accuracy remains limited. Nonetheless, factors such as advanced age and the presence of atrial fibrillation have been consistently associated with an increased risk of major adverse cardiac events in elderly patients undergoing surgery [24].

Respiratory complications also represent a significant concern in the perioperative management of older adults. Respiratory depression and hypoventilation may occur because of the sedative effects of anesthetic agents, particularly in patients with pre-existing respiratory compromise. Because elderly patients often exhibit reduced respiratory reserve, careful monitoring of ventilation and cautious adjustment of anesthetic medications are necessary to reduce the risk of respiratory complications during and after surgery [5].

Neurological complications must also be considered in the geriatric population. Postoperative delirium is one of the most common neurological complications in older adults and may significantly affect postoperative recovery and overall outcomes. Although intraoperative hypotension has been proposed as a potential contributing factor, some studies have not identified a direct association between hypotensive episodes and the development of delirium [25]. Nevertheless, strategies aimed at reducing the incidence of postoperative delirium include comprehensive preoperative cognitive assessment and careful intraoperative management to minimize physiological disturbances [3]. In addition to delirium, elderly patients may also experience prolonged sedation because of age-related alterations in pharmacokinetics and pharmacodynamics. These changes may extend the duration of drug effects and require careful selection and dosing of anesthetic agents [6].

Sedation-related complications also represent an important aspect of perioperative care in elderly patients. Excessive sedation may lead to airway obstruction and central nervous system depression, particularly in individuals with underlying respiratory or neurological conditions. For this reason, anesthetic management should be carefully tailored to minimize these risks. Some studies have suggested that anesthesia care is used more frequently in cataract surgery than in other low-risk procedures. This increased use may reflect the preferences of the ophthalmologist rather than differences in patient characteristics [2].

Several factors have been identified as contributing to the increased risk of perioperative complications in geriatric patients. Advanced age and the presence of multiple comorbidities are among the most significant risk factors. Chronic conditions such as cardiovascular and respiratory diseases may complicate anesthetic management and increase perioperative risk [1,3]. In addition, polypharmacy is highly prevalent in the elderly population and may lead to drug interactions and heightened sensitivity to anesthetic agents. As a result, a thorough preoperative review of the patient’s medication regimen is essential to identify potential interactions and optimize perioperative pharmacological management [6].

Strategies to optimize perioperative outcomes

Preoperative optimization is a fundamental component in improving perioperative outcomes in elderly patients undergoing surgery. Effective management of chronic medical conditions plays a central role in this process. Conditions such as hypertension, diabetes, and cardiovascular diseases are highly prevalent in older adults and must be adequately controlled prior to surgical intervention. Stabilizing these conditions helps reduce perioperative risks and contributes to safer anesthetic management [1,26]. In addition to the control of chronic diseases, careful review of the patient’s medication regimen is essential during preoperative preparation. Adjustments to medications that affect coagulation or cardiovascular function may be necessary to prevent adverse drug interactions and reduce the risk of perioperative complications [5,27]. Another important strategy for preoperative optimization involves the implementation of a comprehensive geriatric assessment. This multidisciplinary evaluation can identify frailty and other clinical risk factors, allowing clinicians to implement targeted interventions that may reduce postoperative complications, including delirium [14,26].

The selection of the appropriate anesthetic technique also represents a key factor in optimizing perioperative outcomes in elderly patients. In many cases, topical or regional anesthesia is preferred over general anesthesia because these approaches are associated with a lower risk profile and exert less impact on cognitive function in older adults. In addition to the choice of anesthetic technique, careful management of sedation is essential. The use of minimal effective doses of sedative agents and the avoidance of deep sedation can help prevent respiratory complications and reduce the likelihood of cognitive dysfunction during the perioperative period [2,28].

Intraoperative monitoring is another critical aspect of perioperative safety. Continuous monitoring of vital signs and respiratory function allows clinicians to promptly identify and address any deviations from physiological stability, particularly when sedation is administered. Close surveillance of these parameters helps reduce the risk of complications and contributes to safer intraoperative management [3,6]. Achieving optimal perioperative care also requires a multidisciplinary approach in which anesthesiologists, ophthalmologists, and perioperative staff collaborate to ensure coordinated patient management and rapid response to potential intraoperative challenges [1,27].

Beyond the intraoperative period, multidisciplinary collaboration remains essential for improving overall patient outcomes. Coordinated teamwork among healthcare professionals, including geriatricians and social workers, can enhance both preoperative preparation and postoperative recovery by addressing the medical as well as the psychosocial needs of elderly patients [26]. In this context, the implementation of enhanced recovery after surgery protocols has been proposed as an effective strategy for improving perioperative outcomes. These protocols aim to promote early mobilization, optimize pain management, and facilitate recovery, thereby contributing to improved postoperative results in elderly patients [29].

## Conclusions

Age-related physiological changes affecting cardiovascular, respiratory, neurological, and pharmacological systems significantly influence anesthetic management in elderly patients undergoing cataract surgery. Reduced cardiac reserve, impaired respiratory function, increased sensitivity to anesthetic agents, and altered pharmacokinetics and pharmacodynamics increase the vulnerability of this population to perioperative complications, highlighting the need for individualized anesthetic strategies and careful dose titration.

Optimizing perioperative outcomes in geriatric patients requires comprehensive preoperative evaluation, appropriate selection of anesthetic techniques, and meticulous intraoperative monitoring. The identification and management of comorbidities, assessment of functional and cognitive status, careful medication review, and the preferential use of topical or regional anesthesia with minimal sedation contribute to improved safety and reduced perioperative risk in elderly individuals undergoing cataract surgery.
